# Status epilepticus during the COVID-19 pandemic in Cologne, Germany: data from a retrospective, multicentre registry

**DOI:** 10.1007/s00415-022-11260-2

**Published:** 2022-07-08

**Authors:** Felix Kohle, Marie Madlener, Emanuel Francesco Bruno, Gereon Rudolf Fink, Volker Limmroth, Lothar Burghaus, Michael Peter Malter

**Affiliations:** 1 Department of Neurology, Faculty of Medicine, University of Cologne and University Hospital of Cologne, Kerpener Strasse, 62, 50937 Cologne, Germany; 2 Department of Neurology and Palliative Medicine, Cologne City Hospitals, Cologne, Germany; 3 Cognitive Neuroscience, Institute of Neuroscience and Medicine (INM-3), Research Centre Juelich, Juelich, Germany; 4 Department of Neurology, Heilig Geist Krankenhaus, Cologne, Germany

**Keywords:** SARS-CoV-2, COVID-19, Seizure, Status epilepticus

## Abstract

**Background:**

The “coronavirus disease 2019” (COVID-19) pandemic, caused by the “severe-acute-respiratory-syndrome-coronavirus 2” (SARS-CoV-2), challenges healthcare systems worldwide and impacts not only COVID-19 patients but also other emergencies. To date, data are scarce on the extent to which the COVID-19 pandemic impacted status epilepticus (SE) and its treatment.

**Objective:**

To assess the influence of the COVID-19 pandemic on the incidence, management and outcome of SE patients.

**Study design:**

This is a retrospective, multicentre trial, approved by the University of Cologne (21-1443-retro).

**Methods:**

All SE patients from the urban area of Cologne transmitted to all acute neurological departments in Cologne between 03/2019 and 02/2021 were retrospectively analysed and assessed for patient characteristics, SE characteristics, management, and outcome in the first pandemic year compared to the last pre-pandemic year.

**Results:**

157 pre-pandemic (03/2019–02/2020) and 171 pandemic (from 03/2020 to 02/2021) SE patients were included in the analyses. Acute SARS-CoV-2 infections were rarely detected. Patient characteristics, management, and outcome did not reveal significant groupwise differences. In contrast, regarding prehospital management, a prolonged patient transfer to the hospital and variations in SE aetiologies compared to the last pre-pandemic year were observed with less chronic vascular and more cryptogenic and anoxic SE cases. No infections with SARS-CoV-2 occurred during inpatient stays.

**Conclusions:**

SARS-CoV-2 infections did not directly affect SE patients, but the transfer of SE patients to emergency departments was delayed. Interestingly, SE aetiology rates shifted, which warrants further exploration. Fears of contracting an in-hospital SARS-CoV-2-infection were unfounded due to consequent containment measures.

**Supplementary Information:**

The online version contains supplementary material available at 10.1007/s00415-022-11260-2.

## Introduction

Status epilepticus (SE) is a medical emergency with high morbidity and mortality and an incidence of annually 10–20 per 100,000 inhabitants in Germany [1]. It is defined by prolonged seizures or a series of seizures with an incomplete return of consciousness in between [2]. The typical therapeutic approach is the immediate SE termination to avoid neuronal damage. The causes for SE include non-adherence of epilepsy patients, cerebrovascular events, drug or alcohol withdrawal, or metabolic disturbances [3].

The “severe-acute-respiratory-syndrome-coronavirus 2” (SARS-CoV-2) is responsible for the latest worldwide pandemic, affecting almost any country in the world and having severe consequences for the overall emergency health care systems [4]. SARS-CoV-2 causes the lung disease “coronavirus disease 2019” (COVID-19) and still poses an international global health challenge [5, 6]. In Germany, the first COVID-19 patient was detected on the 27th of January 2020 [7] and the first lockdown, characterized by severe protection measures, such as closing businesses and schools, the obligation to wear facemasks in public spaces and the restriction of social contacts, occurred from the 22nd of March 2020 to the 4th of May 2020. After its lift, wide-ranging contact restrictions remained in place throughout the summer of 2020. Due to the gradual resurgence of infection rates in the fall 2020, a second lockdown—this time without the closing of schools and most businesses—was imposed the 2nd of November 2020, which lasted until May 2021 [8, 9]. The COVID-19-related social restrictions, as well as its burden on the health care system, resulted in care problems for patients with many other conditions than COVID-19 [10].

For epilepsy patients, many concerns have been discussed concerning the COVID-19 pandemic. A SARS-CoV-2 infection may affect the central nervous system, possibly resulting in seizures and SE [11–14]. In particular, deterioration of seizure control in epilepsy patients was feared because of more difficult access to prescribing physicians [12, 15]. Another concern was a shortage of antiseizure medicaments due to the disruption of global supply chains [16]. Reduced social contacts could worsen epilepsy patient monitoring, resulting in delayed seizure detection [15]. The at times critical overall availability of ICU beds may also have had implications for SE patients. Furthermore, there were also concerns that hospitalization could lead to SARS-CoV-2-infection with subsequent complications [11, 17]. Despite these concerns, data on these issues about SE patients remain scarce.

This study aimed to evaluate subsequent effects of the COVID-19 pandemic on the incidence, management and outcome of SE patients in a representative European urban area in the first year of the COVID 19 pandemic. For this purpose, we initiated the *Project for Status Epilepticus in Cologne* (PROSECO), a scientific association of all neurological departments of Cologne, the fourth largest city in Germany with around one million inhabitants, comprising all acutely admitted SE patients.

## Methods

### Patient selection

All SE patients admitted to one of the three hospitals in Cologne with a neurological department were retrospectively included. The participating hospitals were the University Hospital of Cologne (UHC), Cologne City Hospital Merheim (CCM), and the Heilig Geist-Hospital Cologne (HGH). Altogether, the three hospitals provided care for 10,935 neurological inpatients in 2019 and 10,005 in 2020 according to internal quality reports. As a traffic and touristic hub, Cologne is prone to outbreaks of infectious diseases, such as the COVID-19 pandemic.

All patients included in this study fulfilled the criteria for SE defined by the International League Against Epilepsy (ILAE) with a seizure duration for generalised convulsive SE ≥ 5 min and other SE ≥ 10 min [2]. In the case of general convulsive seizures, the diagnosis was made by typical clinical manifestation, and in the case of non-convulsive SE, the “Salzburg-criteria” were used [18]. Data were extracted from electronic hospital databases. Every patient was rechecked for inclusion and exclusion criteria (for exclusion criteria, see Supplemental material 1).

Inclusion criteria were as follows:

- Patient administered between 03/2019 and 02/2021.
- SE patients admitted directly to one of the neurology departments or by drip-and-ship (i.e., primarily seen in the emergency department of another hospital but directly forwarded).
- Preclinical onset of SE.
- Age ≥ 18 years.
- SE criteria met [2].

### Data collection

All included patients were screened for the following *characteristics before SE*: gender, age, care status, and modified Rankin Scale (mRS) [19, 20]. The following *SE characteristics* were obtained: SE semiology and underlying aetiology according to the epidemiology-based mortality score in status epilepticus (EMSE) [21]. *SE-COVID-19 characteristics,* including an acute SARS-CoV-2-infection and a post-COVID-syndrome, were also assessed. *SE management characteristics* comprised treatment, the onset of SE < 0.5 h until admission, cessation of SE at admission, need and duration of assisted ventilation, duration of ICU and in-hospital stay. *Outcome at discharge* was assessed by the SE cessation rate, mRS, and worsening of mRS compared to the prehospital setting, discharge into self-care, and intrahospital mortality.

### Patient cohorts

To assess whether the COVID-19 pandemic had any direct or indirect influence on SE management, patients admitted between 03/2019 and 02/2020 served as the pre-pandemic group (pre-COV), whereas patients admitted between 03/2020 and 02/2021 were grouped as the pandemic group (COV).

### Statistical analysis

The data analyses were performed using SSPS software 28.0 for Windows (IBM, Armonk, New York, USA). For comparisons of independent categorical data, chi-square tests or Fisher’s exact tests (if less than 5 items) were performed; for comparisons of independent metrical data, *t* tests for unpaired variables were performed. All tests were performed two-tailed. A *p*-value < 0.05 was considered significant.

## Results

### Selection process

**Fig. 1:**
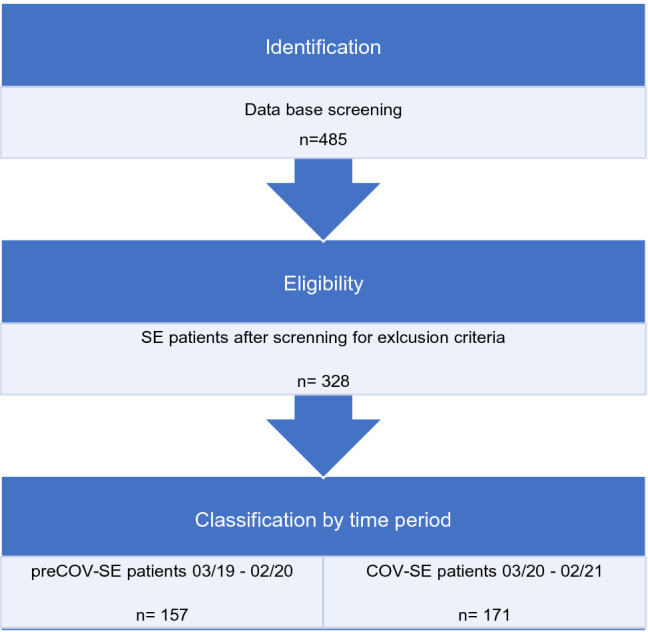
Selection process of status epilepticus (SE) patients for analysis inclusion. Overall, 485 status epilepticus (SE) patients were identified by database screening. 157 patients met one or more exclusion criteria. 157 SE patients, admitted between 03/2019 and 02/2020 to a neurological department in Cologne, served as the control group (preCOV), and 171 SE patients between 03/2020 and 02/2021 constituted the pandemic SE group (COV)

Our multicentre data base search resulted in a total of 485 patients. Inclusion criteria were fulfilled in 328 patients, with 157 patients in the pre-COV group and 171 patients in the COV group. Overall, 157 patients had to be excluded. 89 patients did not meet ILAE-criteria for SE, and five patients developed SE during an inpatient stay. Nine patients were not treated in neurological departments and were excluded. Another 54 patients were admitted from hospitals outside the urban area of Cologne or developed SE outside the urban area of Cologne (see Fig. 1).

### Overall characteristics, management and outcome at discharge

Of the 328 SE patients analysed, 214 patients were treated at the UHC (65.2%), 71 (21.6%) at the CCM, and 43 (13.1%) at the HGH. 96.6% of the patients were direct admissions. Epilepsy was known in 213 patients (64.9%). 142 of all patients had a generalised convulsive (GC) semiology (43.3%). 264 SE patients had a chronic SE aetiology (80.5%), while acute-symptomatic SE occurred in 37 patients (11.3%). SE aetiology remained unknown in 23 cases (7%). In four patients, the SE aetiology could be attributed to both acute-symptomatic SE and chronic SE aetiology.

The functional outcome according to mRS worsened in 73 patients (22.3%). Overall, 20 patients died in-hospital, resulting in an overall intrahospital mortality rate of 6.1%. Six patients were discharged into palliative care, three with a persistent SE and three due to other comorbidities. Including these patients, the adjusted mortality rate rises to 7.9%. In 16 patients (4.9%), SE was never breached. In four patients (1.2%), the data were inconclusive whether SE was breached or not.

The respective details are given in Table 1.

**Table 1:** Overall characteristics of all status epilepticus (SE) patients

Patient characteristics	
Female, *n* (%)	150 (45.7%)
Median age in years (SD, range)	66.4 (18.8, 18.4–93.5)
Pre-pandemic, *n* (%)	157
Pandemic, *n* (%)	171
Admitted to, *n* (%)	
UHC	214 (65.2%)
HGH	43 (13.1%)
CCM	71 (21.6%)
Direct admission, *n* (%)	317 (96.6%)
Admitted from home, *n* (%) Admitted from nursing home, *n* (%)	233 (71.0%) 95 (29.0%)
Known epilepsy, *n* (%)	213 (64.9%)
Median mRS, (SD, range) before admission	3 (1.8, 0–5)
SE characteristics	
GC semiology, n (%)	142 (43.3%)
Median STESS Score, (SD, range) [22]	2 (1.5, 0–6)
Median EMSE Score, (SD, range) [21]	44 (30.5, 2–188)
Aetiology of SE, *n* (%)	
Acute	37 (11.3%)
Chronic	264 (80.5%)
Cryptogenic	23 (7%)
Acute and chronic	4 (1.2%)
Aetiology of SE detailed (according to EMSE), *n* (%)	
CNS anomaly	17 (5.2%)
Drug withdrawal/incompliance	37 (11.3%)
Multiple sclerosis	7 (2.1%)
Chronic cerebrovascular disease	133 (40.5%)
Hydrocephalus	2 (0.6%)
Alcohol abuse	7 2(0.1%)
Drug intoxication	1 (0.3%)
Acute cerebral injury	1 (0.3%)
Cryptogenic	23 (7.0%)
Brain tumour	37 (11.3%)
Sodium disturbance	7 (2.1%)
Other metabolic disturbance	6 (1.8%)
Acute cerebrovascular disease	5 (1.5%)
Acute CNS infection	3 (0.9%)
Anoxia	9 (2.7%)
Autoimmune encephalitis	3 (0.9%)
Epilepsy without provocation	5 (1.5%)
Others	25 (7.6%)
COVID-19	
SARS-CoV-2 positive, *n* (%)	3 (0.9%)
Post-COVID syndrome, *n* (%)	0 (0%)
Management of SE	
Initial benzodiazepine therapy, *n* (%)	236 (72.0%)
Median number of drugs (SD, range)	2 (1.4, 0–7)
SE onset < 0.5 h at arrival, *n* (%)	65 (19.8%)
SE ceased at hospital arrival, *n* (%)	69 (21%)
ICU admission, *n* (%)	247 (75.3%)
Mechanical ventilation, *n* (%)	49 (14.9%)
Median duration of ventilation in hours (SD, range)	54.6 (210, 0–921)
Median duration ICU stay in days (SD, range)	1.3 (6.6, 0–63.2)
Median duration in-hospital stay in days (SD, range)	4.3 (11.0, 0.1–103.9)
Outcome at discharge	
SE remitted at discharge, *n* (%)	308 (93.9%)
Median mRS, (SD, range) at discharge	3 (2.0, 0.6)
MRS worsened at discharge, *n* (%)	73 (22.3)
Discharge at home, *n* (%)	251 (76.5)
Lethal outcome	20 (6.1)

Detailed are patient and SE characteristics, the “coronavirus disease 2019” (COVID-19) characteristics, and the management and outcome of the SE patients who were admitted to the participating neurological hospitals forming the *Project for Status Epilepticus in Cologne* (PROSECO) between 03/2019 and 02/2022

UHC, University Hospital of Cologne; CCM, Cologne City Hospital Merheim; HGH, Heilig Geist-Hospital Cologne; mRS, modified Rankin scale; GC, generalised convulsive; STESS, status epilepticus severity score; EMSE, epidemiology-based mortality score in status epilepticus; SARS-CoV-2, severe acute respiratory syndrome coronavirus 2; ICU, intensive care unit. Standard deviation (SD) is provided where appropriate

### Pandemic characteristics

Only three (2%) of the 171 SE patients admitted during the first pandemic year were SARS-CoV-2 positive. Two of them had COVID-19-related signs, such as coughing and fever, and one was asymptomatic. None of the patients in the COV group was diagnosed with post-COVID-syndrome.

The overall patients’ characteristics showed no differences in the COV and preCOV groups (see Table 2).

**Table 2:** Comparison of status epilepticus (SE) patients in the prepandemic year (preCOV) to the patients of the first year of the “coronavirus disease 2019” (COVID-19) pandemic (COV)

	Pre-COV (*N* = 157) *N* (%)	COV (*N* = 171) *N* (%)	Significance
Patient characteristics			
Female, *n* (%)	70 (44.6)	80 (46.8)	0.74
Mean age in years (SD, range)	64.2 (19.0)	62.7 (18.7)	0.46
Admitted hospital, *n* (%)			
UHC	101 (64.3)	113 (66.1)	0.92
HGH	22 (14.0)	21 (12.3)	
UCM	34 (21.7)	37 (21.6)	
Direct admission, *n* (%)	151 (96.2)	166 (97.1)	0.76
Living at home, *n* (%)	107 (68.2)	126 (73.7)	0.28
Know epilepsy, *n* (%)	97 (61.8)	116 (67.8)	0.30
Mean mRS (SD) before admission	2.6 (1.7)	2.5 (1.8)	0.4
SE characteristics			
GC semiology, *n* (%)	62 (39.5)	80 (46.8)	0.22
Aetiology of SE, *n* (%)			
Acute	17 (10.8)	20 (11.7)	**0.02**
Chronic	134 (85.4)	130 (76.0)	
Cryptogenic	5 (3.2)	18 (10.5)	
Acute and chronic	1 (0.6)	1 (1.8)	
Mean STESS Score, (SD) [22]	2.5 (1.5)	2.4 (1.4)	0.5
Mean EMSE Score, (SD) [21]	47.0 (29.5)	51.3 (31.3)	0.2
Aetiology of SE detailed, *n* (%)			
CNS anomaly	9 (5.7)	8 (4.7)	0.80
Drug withdrawal/incompliance	19 (12.1)	18 (10.5)	0.73
Multiple sclerosis	2 (1.3)	5 (2.9)	0.45
Chronic cerebrovascular disease	82 (52.2)	51 (29.8)	** < 0.01**
Hydrocephalus	1 (0.6)	1 (0.6)	1
Alcohol abuse	4 (2.5)	3 (1.8)	0.71
Drug intoxication	1 (0.6)	0 (0)	0.48
Acute cerebral injury	1 (0.6)	0 (0)	0.48
Cryptogenic	5 (3.2)	18 (10.5)	**0.01**
Brain tumour	14 (8.9)	23 (13.5)	0.29
Sodium disturbance	2 (1.3)	5 (2.9)	0.45
Other metabolic disturbance	4 (2.5)	2 (1.2)	0.43
Acute cerebrovascular disease	1 (0.6)	4 (2.3)	0.37
Acute CNS infection	1 (0.6)	2 (1.2)	1
Anoxia	1 (0.6)	8 (4.7)	**0.04**
Autoimmune encephalitis	1 (0.6)	2 (1.2)	1
Epilepsy without provocation	3 (1.9)	2 (1.2)	0.67
Others	6 (3.8)	19 (11.1)	**0.02**
Management of SE			
Initial benzodiazepine, *n* (%)	113/153 (73.9)	123/156 (78.8)	0.35
Mean number of drugs (SD, range)	2.5 (1.5)	2.4 (1.4)	0.12
SE onset < 0.5 h at arrival, *n* (%)	39 (24.8)	26 (15.2)	**0.04**
SE ceased at hospital arrival, *n* (%)	28 (17.8)	41 (24.0)	0.2
ICU admission, *n* (%)	126 (80.3)	121 (70.8)	0.55
Mechanical ventilation, *n* (%)	23 (14.6)	26 (15.2)	1
Mean duration of ventilation in hours (SD, range)	147.4 (228.6)	136.1 (196.4)	0.85
Mean duration ICU stay in days (SD, range)	3.0 (6.5)	3.3 (6.8)	0.36
Mean duration in-hospital stay in days (SD, range)	7.9 (11.3)	6.8 (10.7)	0.36
Outcome at discharge			
SE remitted at discharge, *n* (%)	151 (96.8)	157 (93.5)	0.20
MRS, (SD, range) at discharge	2.9 (1.8)	2.9 (2.0)	1
MRS worsened at discharge, *n* (%)	28 (17.8)	45 (26.3)	0.08
Discharge at home, *n* (%)	117 (74.5)	134 (78.4)	0.44
Lethal outcome	6 (3.8)	14 (8.2)	0.1

*p*-values <0.05 (stastically significant) are shown in bold

Detailed are patient and SE characteristics and the management and outcome of the SE patients

UHC, University Hospital of Cologne; CCM, Cologne City Hospital Cologne—Merheim; HGH, Heilig Geist-Hospital Cologne; mRS, modified Rankin scale; GC, generalised convulsive; STESS, status epilepticus severity score; EMSE, epidemiology-based mortality score in status epilepticus; ICU, intensive care unit. Standard deviations (SD) are provided where appropriate

However, we could observe significant differences in the SE characteristics. The number of patients who arrived at the hospital < 0.5 h after SE onset decreased from 24.8% preCOV to 15.2% COV (*p*-value: 0.04). There was a significant increase of cryptogenic SE aetiology from 3.2% preCOV to 10.5% COV (*p*-value: 0.02) and a corresponding decrease of patients with chronic SE aetiology (85.4% preCOV to 76.0% COV). By detailed assessment of the aetiology using the EMSE classification, we remarked a significant decrease in SE patients with chronic cerebrovascular diseases (52.2% preCOV compared to 29.8% COV, *p*-value < 0.01). Remarkably, anoxia as underlying aetiology increased also significantly during the pandemic (0.6% preCOV to 4.7% COV, *p*-value: 0.04).

Management of SE and outcome at discharge did not reveal differences between preCOV and COV patients. Details are given in Table 2.

## Discussion

One key finding of our study of SE patients in Cologne in the first year of the COVID-19 pandemic is that an acute SARS-CoV-2 infection was rare. Furthermore, SE incidence did not increase in patients with previously diagnosed epilepsy. As expected, the patient transfer time to a neurological department was significantly delayed. Interestingly, we observed a shift in the SE aetiologies. The fear of acquiring a SARS-CoV-2 infection during the in-hospital stay was not justified.

SE incidence ranges from 5 to 41 per 100.000 adults [1, 22–24]. Overall, 328 patients with confirmed SE were admitted to the neurological departments in Cologne. In the years under investigation, approximately 914.410 adult inhabitants lived in this study’s catchment area [25], resulting in an incidence of 17.9 per 100.000 adults per year. As in other studies, slightly fewer females than males (45.7–54.3%) suffered from SE [1, 23]. SE risk increases with age; the median age was 66.4 years in our study. One reason for the increasing incidence with age is the higher prevalence of chronic cerebrovascular disease, which is the most typical SE aetiology in our study (40.5%) [22, 26, 27]. The overall mortality rate of 6.1% in our study is remarkably lower than previously reported mortality rates of around 20% [28, 29], even when adjusting the rate to include patients directly transmitted into palliative care (the mortality-rate increases to 7.9%). Possible explanations for differences in the mortality rates are different evaluation parameters, e.g., inpatient fatality [28] vs. 30-day mortality [3]. Besides, changes in the ILAE classification in 2015 increased the incidence of SE by about 10% [28] and the higher incidence of convulsive SE of 43.3% in our study (compared to 36.1% to the epidemiological study from Salzburg), associated with an improved prognosis compared to non-convulsive SE, could explain the observed differences [28]. In line with our data, a comparatively low 30-day mortality rate of 4.6% was found in a population-based study of SE outcome in Auckland, in which 81% of the patients showed a convulsive semiology [30].

Overall, our epidemiological data are in line with previously reported SE characteristics [1, 3, 22, 23, 28, 30], enabling detailed analyses of the impact of the COVID-19 pandemic on SE incidence, management, and outcome.

Besides a few case reports and series, a SARS-CoV-2-infection itself does not cause seizures, while neurological complications of COVID-19 are known triggers [17, 31]. In line with this, only 2% of all SE patients suffered from a SARS-CoV-2 infection, indicating that acute SARS-CoV-2 infections and COVID-19 complications played no significant role in the SE management in the first pandemic year. The small number of patients with concurrent SARS-CoV-2 infection is comparable to the positive cases registered by the Robert-Koch Institute (responsible for notifiable infectious diseases in Germany). In our study’s observation period, 34.737 proven infections were reported in adults in Cologne (3.8%) (personal communication from the Landeszentrum Gesundheit Nordrhein-Westfalen, Public Health Department in North Rhine-Westphalia). Since all patients admitted to a hospital in Germany were screened for SARS-CoV-2 infections at admission by PCR testing, we do not assume an underreporting in our cohort. In sum, we did not find that an active SARS-CoV-2-infection or post-COVID-syndrome caused SE or impacted SE management directly.

For the first year of the COVID-19 pandemic, the overall SE incidence rates and epidemiological characteristics did not change significantly. The findings align with a recent comparative retrospective study from Austria, which compared the characteristics of a first SE in the first 2 months of the pandemic to a corresponding pre-pandemic period, which could not observe any significant difference in the SE incidence [32]. Contrary to previous assumptions [12, 15], drug withdrawal or reduced access to physicians causing SE were not more prominent in the first pandemic year. Another retrospective study, which analysed prospective data of a large epilepsy cohort in Germany, found that adherence to antiseizure medication remained stable during the first lockdown [33]. In Italy, a survey by the ILAE-COVID-19 and Telemedicine Task Forces of caregivers showed that 22.8% of patients with epilepsy and 27.5% of caregivers reported an increase in seizure frequency during the pandemic [34, 35]. Data may not be directly comparable between Germany and Italy due to differences in care structures and health care burden during the pandemic. In sum, we did not observe that the COVID-19 pandemic impacted SE incidence in patients with known epilepsy for the urban area of Cologne.

A critical difference between the preCOV and COV period was the significantly prolonged patient transfer time to neurological emergency departments. The percentage of patients with SE who arrived at < 0.5 h after SE onset at one of the neurological emergency departments in Cologne decreased significantly from 24.8 to 15.2% in the first pandemic year. Besides the underlying cause, one essential factor in SE outcome is immediate treatment initiation [24]. Although detailed information for the delayed transfer is lacking, an overload of the emergency services or delayed alerting of the emergency services seem plausible. Reasons for the latter could have been contact restrictions with delayed SE recognition or fears of hospitalisation with the risk of acquiring a SARS-CoV-2 infection.

We observed significant shifts in the SE aetiologies during the first pandemic year (see Table 2). There was a significant increase of cryptogenic aetiologies from 3.2% to 10.5%. Causes for this observation remain elusive. As only three SE patients were SARS-CoV-2 positive, we cannot see a direct connection to COVID-19, although indirect effects cannot be excluded.

Remarkably, patients with SE due to chronic cerebrovascular diseases decreased significantly (52.2% preCOV compared to 29.8% COV). It is well known that stroke admissions diminished during lockdowns in many countries [10, 36]. An analysis of the nationwide German stroke cohort study found a significant decrease in the numbers for both ischemic and haemorrhage strokes in Germany [10]. The reduction was particularly significant for patients with transient ischemic attacks who may have decided not to seek medical care for fear of in-hospital SARS-CoV-2-infection. Alternatively, urgently needed hospital capacities may have been limited [11]. In line with the latter, mechanical thrombectomy rates slightly increased in this period, suggesting that prominent and acute neurological motor and language deficits led to undebated hospital admissions even during periods of contact restrictions [10]. Consistently, the rate of SE caused by acute cerebrovascular disease did not change in the first pandemic year.

Furthermore, anoxia as a SE aetiology increased significantly during the first pandemic year from 0.6 to 4.7%. The low incidence of acute SARS-CoV-2-infections with only three patients does not suggest that the higher incidence results from COVID-19 leading to hypoxia.

Notably, the observed shift in SE aetiology cannot be ascribed to changes in the diagnostic work-up (see Supplemental material 2).

A putative fear of acquiring a SARS-CoV-2 infection through hospitalisation was unfounded in our patient population. Patients in Cologne with concurrent COVID-19 and neurological disorders were triaged according to their life-threatening problem and referred to the corresponding ICU. Suspected SARS-CoV-2 infected patients were separated from other patients by established pathways in the accident and emergency departments. High-risk patients were isolated until a negative test result was obtained. None of the SE patients developed a SARS-CoV-2 infection during the inpatient stay. Similar results were reported in an observational study from the German Heart Center in Berlin, in which 589 patients and 394 hospital employees were assessed for SARS-CoV-2 infections via PCR-testing, resulting in no nosocomial-acquired infection [37]. This effect is likely due to rigorous PCR screening of all patients for SARS-CoV-2.

The retrospective design bears known inherent limitations. Reasons for the delayed patient transfer could not be determined from the records. Due to the different nature of the pandemic dynamics in each country with different political and local handling approaches and varying health care systems, general conclusions should be drawn with caution [32].

In summary, our study showed that the COVID-19 pandemic did not increase SE incidence but significantly prolonged patient transfer time to neurological emergency departments. The SE aetiology shifted from chronic vascular to cryptogenic and anoxic causes during the first year of the pandemic, while the fear of an hospital-acquired SARS-CoV-2-infection was not justified in our cohort due to rigorous containment measures.

## Supplementary Information

Below is the link to the electronic supplementary material.

Supplementary file1 (DOCM 16 kb)

## Abbreviations

CCM: City Hospital Cologne-Merheim
COVID-19: Coronavirus disease 2019
CSF: Cerebrospinal fluid
EEG: Electroencephalography
EMSE: Epidemiology-based mortality score in status epilepticus
GC: Generalized-tonic convulsive
HGH: Heilig Geist-Hospital Cologne
ICU: Intensive care unit
ILAE: International League Against Epilepsy
MRI: Magnetic resonance imaging
mRS: Modified Rankin scale
PCR: Polymerase chain reaction
PROSECO: Project for Status Epilepticus in Cologne
PWE: Persons with epilepsy
SARS-CoV-2: Severe acute respiratory syndrome coronavirus 2
SD: Standard deviation
SE: Status epilepticus
STESS: Status epilepticus severity score
UHC: University Hospital of Cologne

## References

[1] Knake S, Rosenow F, Vescovi M, Oertel WH, Mueller HH, Wirbatz A, Katsarou N, Hamer HM (2001). Incidence of status epilepticus in adults in Germany: a prospective, population-based study. Epilepsia.

[2] Trinka E, Cock H, Hesdorffer D, Rossetti AO, Scheffer IE, Shinnar S, Shorvon S, Lowenstein DH (2015). A definition and classification of status epilepticus—report of the ILAE Task Force on Classification of Status Epilepticus. Epilepsia.

[3] DeLorenzo RJ, Hauser WA, Towne AR, Boggs JG, Pellock JM, Penberthy L, Garnett L, Fortner CA, Ko D (1996). A prospective, population-based epidemiologic study of status epilepticus in Richmond, Virginia. Neurology.

[4] Dong E, Du H, Gardner L (2020). An interactive web-based dashboard to track COVID-19 in real time. Lancet Infect Dis.

[5] Zhu N, Zhang D, Wang W, Li X, Yang B, Song J, Zhao X, Huang B, Shi W, Lu R, Niu P, Zhan F, Ma X, Wang D, Xu W, Wu G, Gao GF, Tan W (2020). A novel coronavirus from patients with pneumonia in China, 2019. N Engl J Med.

[6] Huang C, Wang Y, Li X, Ren L, Zhao J, Hu Y, Zhang L, Fan G, Xu J, Gu X, Cheng Z, Yu T, Xia J, Wei Y, Wu W, Xie X, Yin W, Li H, Liu M, Xiao Y, Gao H, Guo L, Xie J, Wang G, Jiang R, Gao Z, Jin Q, Wang J, Cao B (2020). Clinical features of patients infected with 2019 novel coronavirus in Wuhan, China. Lancet.

[7] Streeck H, Schulte B, Kümmerer BM, Richter E, Höller T, Fuhrmann C, Bartok E, Dolscheid-Pommerich R, Berger M, Wessendorf L, Eschbach-Bludau M, Kellings A, Schwaiger A, Coenen M, Hoffmann P, Stoffel-Wagner B, Nöthen MM, Eis-Hübinger AM, Exner M, Schmithausen RM, Schmid M, Hartmann G (2020). Infection fatality rate of SARS-CoV2 in a super-spreading event in Germany. Nat Commun.

[8] Karagiannidis C, Windisch W, McAuley DF, Welte T, Busse R (2021). Major differences in ICU admissions during the first and second COVID-19 wave in Germany. Lancet Respir Med.

[9] Schuppert A, Polotzek K, Schmitt J, Busse R, Karschau J, Karagiannidis C (2021). Different spreading dynamics throughout Germany during the second wave of the COVID-19 pandemic: a time series study based on national surveillance data. Lancet Reg Health Eur.

[10] Richter D, Eyding J, Weber R, Bartig D, Grau A, Hacke W, Krogias C (2021). Analysis of nationwide stroke patient care in times of COVID-19 pandemic in Germany. Stroke.

[11] Aguiar de Sousa D, Sandset EC, Elkind MSV (2020). The curious case of the missing strokes during the COVID-19 pandemic. Stroke.

[12] Berlit P, Bösel J, Gahn G, Isenmann S, Meuth SG, Nolte CH, Pawlitzki M, Rosenow F, Schoser B, Thomalla G, Hummel T (2020). "Neurological manifestations of COVID-19"—guideline of the German society of neurology. Neurol Res Pract.

[13] Kim HK, Cho YJ, Lee SY (2021). Neurological manifestations in patients with COVID-19: experiences from the central infectious diseases hospital in South Korea. J Clin Neurol.

[14] Asadi-Pooya AA, Simani L, Shahisavandi M, Barzegar Z (2021). COVID-19, de novo seizures, and epilepsy: a systematic review. Neurol Sci.

[15] French JA, Brodie MJ, Caraballo R, Devinsky O, Ding D, Jehi L, Jette N, Kanner A, Modi AC, Newton CR, Patel AA, Pennell PB, Perucca E, Sander JW, Scheffer IE, Singh G, Williams E, Wilmshurst J, Cross JH (2020). Keeping people with epilepsy safe during the COVID-19 pandemic. Neurology.

[16] Albert DVF, Das RR, Acharya JN, Lee JW, Pollard JR, Punia V, Keller JA, Husain AM (2020). The Impact of COVID-19 on epilepsy care: a survey of the American epilepsy society membership. Epilepsy Curr.

[17] Belluzzo M, Nilo A, Valente M, Gigli GL (2021). New-onset status epilepticus in SARS-CoV-2 infection: a case series. Neurol Sci.

[18] Beniczky S, Hirsch LJ, Kaplan PW, Pressler R, Bauer G, Aurlien H, Brøgger JC, Trinka E (2013). Unified EEG terminology and criteria for nonconvulsive status epilepticus. Epilepsia.

[19] Rankin J (1957). Cerebral vascular accidents in patients over the age of 60: II. Prognosis. Scottish Med J.

[20] van Swieten JC, Koudstaal PJ, Visser MC, Schouten HJ, van Gijn J (1988). Interobserver agreement for the assessment of handicap in stroke patients. Stroke.

[21] Leitinger M, Höller Y, Kalss G, Rohracher A, Novak HF, Höfler J, Dobesberger J, Kuchukhidze G, Trinka E (2015). Epidemiology-based mortality score in status epilepticus (EMSE). Neurocrit Care.

[22] Lv R-J, Wang Q, Cui T, Zhu F, Shao X-Q (2017). Status epilepticus-related etiology, incidence and mortality: a meta-analysis. Epilepsy Res.

[23] Hesdorffer DC, Logroscino G, Cascino G, Annegers JF, Hauser WA (1998). Incidence of status epilepticus in Rochester, Minnesota, 1965–1984. Neurology.

[24] Betjemann JP, Lowenstein DH (2015). Status epilepticus in adults. Lancet Neurol.

[25] Koeln S (2021) Bevölkerungszahl sinkt leicht auf 1 088 040 in 2020. Available from: https://www.stadt-koeln.de/mediaasset/content/pdf15/statistik-einwohner-und-haushalte/kurzinformation_bev%C3%B6lkerung_3_2021.pdf

[26] Bhalla D, Tchalla AE, Mignard C, Marin B, Mignard D, Jallon P, Preux P-M (2014). First-ever population-based study on status epilepticus in French Island of La Reunion (France)—incidence and fatality. Seizure.

[27] Govoni V, Fallica E, Monetti VC, Guerzoni F, Faggioli R, Casetta I, Granieri E (2008). Incidence of status epilepticus in Southern Europe: a population study in the Health District of Ferrara, Italy. Eur Neurol.

[28] Leitinger M, Trinka E, Giovannini G, Zimmermann G, Florea C, Rohracher A, Kalss G, Neuray C, Kreidenhuber R, Höfler J, Kuchukhidze G, Granbichler C, Dobesberger J, Novak HF, Pilz G, Meletti S, Siebert U (2019). Epidemiology of status epilepticus in adults: a population-based study on incidence, causes, and outcomes. Epilepsia.

[29] DeLorenzo RJ, Pellock JM, Towne AR, Boggs JG (1995). Epidemiology of status epilepticus. J Clin Neurophysiol.

[30] Bergin PS, Brockington A, Jayabal J, Scott S, Litchfield R, Roberts L, Timog J, Beilharz E, Dalziel SR, Jones P, Yates K, Thornton V, Walker EB, Davis S, Te Ao B, Parmar P, Beghi E, Rossetti AO, Feigin V (2019). Status epilepticus in Auckland, New Zealand: incidence, etiology, and outcomes. Epilepsia.

[31] Pauletto G, Nilo A, Deana C, Verriello L, Del Negro I, Lettieri C, Vetrugno L, Valente M, Gigli GL (2021). Recurrent status epilepticus and SARS-CoV-2 infection: the "perfect storm". Acta Biomed.

[32] Leitinger M, Poppert KN, Mauritz M, Rossini F, Zimmermann G, Rohracher A, Kalss G, Kuchukhidze G, Höfler J, Bosque Varela P, Kreidenhuber R, Volna K, Neuray C, Kobulashvili T, Granbichler CA, Siebert U, Trinka E (2020). Status epilepticus admissions during the COVID-19 pandemic in Salzburg-A population-based study. Epilepsia.

[33] Mueller TM, Kostev K, Gollwitzer S, Lang JD, Stritzelberger J, Westermayer V, Reindl C, Hamer HM (2021). The impact of the coronavirus disease (COVID-19) pandemic on outpatient epilepsy care: an analysis of physician practices in Germany. Epilepsy Behav.

[34] Cross JH, Kwon CS, Asadi-Pooya AA, Balagura G, Gómez-Iglesias P, Guekht A, Hall J, Ikeda A, Kishk NA, Murphy P, Kissani N, Naji Y, Perucca E, Pérez-Poveda JC, Sanya EO, Trinka E, Zhou D, Wiebe S, Jette N (2021). Epilepsy care during the COVID-19 pandemic. Epilepsia.

[35] Assenza G, Lanzone J, Brigo F, Coppola A, Di Gennaro G, Di Lazzaro V, Ricci L, Romigi A, Tombini M, Mecarelli O (2020). Epilepsy care in the time of COVID-19 Pandemic in Italy: risk factors for seizure Worsening. Front Neurol.

[36] Kansagra AP, Goyal MS, Hamilton S, Albers GW (2020). Collateral effect of Covid-19 on stroke evaluation in the United States. N Engl J Med.

[37] Schöppenthau D, Weiß KJ, Estepa-Martinez M, Hommel M, Miera O, Schoenrath F, Hübler S, Obermeier M, Pieske B, Stawowy P (2021). Preventing SARS-CoV-2 in-hospital infections in cardiovascular patients and medical staff: an observational study from the German Heart Center Berlin. Front Med.

