# Supplementary material for: Status epilepticus during the COVID-19 pandemic in Cologne, Germany: data from a retrospective, multicentre registry
Source: J Neurol. 2022 Jul 8;269(11):5710–9. doi: 10.1007/s00415-022-11260-2 (PMC9266085; doi:10.1007/s00415-022-11260-2)
Supplement: Supplementary file 1 — Supplementary file1 (DOCM 16 kb) [file 415_2022_11260_MOESM1_ESM.docm]

**SUPPLEMENTAL MATERIAL 1**
Exclusion criteria:

- Patient administered before 03/2019 or after 02/2021
- SE criteria not met
- Non-preclinical SE onset
- Age < 18 years
- Transferred inpatients from other hospitals
- Transfer from hospitals outside the urban area of Cologne
- Inpatient treatment in a non-neurological department of the three participating hospitals

**SUPPLEMENTAL MATERIAL 2**

|  | **All (N=27)** | **PreCOV (N=6)** | **COV (N=21)** | **Significances** |
| --- | --- | --- | --- | --- |
| Cerebral imaging, n (%)  CT, n (%)  MRI, n (%) | 26 (93.3)  11 (40.7)  15 (55.6) | 6 (100)  3 (50)  3 (50) | 20 (95.2)  8 (38.1)  12 (57.1) | 1 |
| EEG, n (%) | 25 (92.6) | 6 (100) | 19 (90.5) | 1 |
| CSF, n (%) | 12 (44.4) | 2 (33.3) | 10 (47.6) | 0.7 |

**Table 1** shows the work-up flow, consisting of cerebrospinal fluid analysis, electroencephalogram (EEG) and head imaging (computed tomography scan or magnetic resonance imaging) of all cryptogenic status epilepticus (SE) patients pre-pandemic (preCOV) and during the first year of the pandemic (COV).
